# Liposomal Formulations: A Recent Update

**DOI:** 10.3390/pharmaceutics17010036

**Published:** 2024-12-30

**Authors:** Surendra S. Agrawal, Vrinda Baliga, Vaishali Y. Londhe

**Affiliations:** 1 Datta Meghe College of Pharmacy, Datta Meghe Institute of Higher Education and Research (DU), Sawangi (M), Wardha 442001, Maharashtra, India; surendra.pharmacy@dmiher.edu.in; 2 Shobhaben Pratapbhai Patel School of Pharmacy & Technology Management, SVKM’s NMIMS, V.L. Mehta Road, Vile Parle (W), Mumbai 400056, Maharashtra, India

**Keywords:** liposomes, advanced technologies, enhancing drug loading capacity, surface modification, preclinical and clinical studies

## Abstract

Liposome-based drug delivery technologies have showed potential in enhancing medication safety and efficacy. Innovative drug loading and release mechanisms highlighted in this review of next-generation liposomal formulations. Due to poor drug release kinetics and loading capacity, conventional liposomes have limited clinical use. Scientists have developed new liposomal carrier medication release control and encapsulation methods to address these limits. Drug encapsulation can be optimized by creating lipid compositions that match a drug’s charge and hydrophobicity. By selecting lipids and adding co-solvents or surfactants, scientists have increased drug loading in liposomal formulations while maintaining stability. Nanotechnology has also created multifunctional liposomes with triggered release and personalized drug delivery. Surface modification methods like PEGylation and ligand conjugation can direct liposomes to disease regions, improving therapeutic efficacy and reducing off-target effects. In addition to drug loading, researchers have focused on spatiotemporal modulation of liposomal carrier medication release. Stimuli-responsive liposomes release drugs in response to bodily signals. Liposomes can be pH- or temperature-sensitive. To improve therapeutic efficacy and reduce systemic toxicity, researchers added stimuli-responsive components to liposomal membranes to precisely control drug release kinetics. Advanced drug delivery technologies like magnetic targeting and ultrasound. Pro Drug, RNA Liposomes approach may improve liposomal medication administration. Magnetic targeting helps liposomes aggregate at illness sites and improves drug delivery, whereas ultrasound-mediated drug release facilitates on-demand release of encapsulated medicines. This review also covers recent preclinical and clinical research showing the therapeutic promise of next-generation liposomal formulations for cancer, infectious diseases, neurological disorders and inflammatory disorders. The transfer of these innovative liposomal formulations from lab to clinical practice involves key difficulties such scalability, manufacturing difficulty, and regulatory limits.

## 1. Introduction

Liposomes have been one of the main formulation types for the development of drug delivery technologies because of their demonstrated ability to encapsulate drug molecules and facilitate safe and efficient medication delivery [1]. Liposomes consist of one or more phospholipid bilayers that can encapsulate both hydrophilic and hydrophobic drugs. Hydrophilic drugs are typically enclosed in the aqueous core, while hydrophobic drugs are trapped within the lipid bilayer. The performance of liposomes in drug delivery is defined by their ability to control the drug release and also ability to target certain cells or tissues. Liposomes shield the medication from removal and destruction giving prolonged therapeutic action [1,2,3]. Liposomes are approved for use in numerous therapeutic applications, including cancer treatment and gene therapy. Different medicinal agents, such as proteins, nucleic acids, and tiny molecules, have been administered through their utilization [4]. The versatility of liposomes accounts for their widespread application, which includes research on vaccines, food and cosmetics, medicinal sectors, and cutaneous and transdermal distribution. This review outlines novel strategies such as surface modification, ultrasound-mediated drug release, stimuli-responsive liposomal formulations, magnetic targeting, and light-responsive liposomes, in addition to the challenges faced by traditional liposomal delivery systems. It covers the challenges faced during the formulation process and the current status of preclinical and clinical studies pertaining to next-generation liposomes [5].

The Prisma S-extension with appropriate mesh terms like liposomes, surface modified liposomes, liposomes for improved drug loading and release, preclinical study and clinical study on liposomes for cancer therapy, liposomes for neurological disorders, infectious disease and inflammatory disorders, and RNA liposomes were followed for obtaining the desired references from PubMed and Scopus. The Boolean search revealed a total of 1860 articles, which was further reduced to 155 after the removal of duplicate records.

## 2. Traditional Liposomal Formulations: Challenges

Physical instability in liposomal formulations can cause drugs to leak, fuse, or agglomerate. Temperature, mechanical strain, and biological fluid interaction can all lead to physical instability. Alterations in the size and surface properties can influence the safety and effectiveness of liposomes [6]. Typical problems associated with the chemical stability of liposomes include lipid oxidation and hydrolysis. This is due to their propensity for oxidation; unsaturated lipids in the liposome bilayer generate reactive oxygen species that are detrimental to the liposome. The release of free fatty acids from the hydrolysis of ester bonds in phospholipids has the potential to disrupt the liposomal membrane.

Following administration, liposomes interact with bodily fluids, which makes them detectable and opsonized by the reticuloendothelial system (RES) [7,8,9]. As a result, soon after entering the bloodstream, the encapsulated medication loses its therapeutic potency. The “accelerated blood clearance” phenomenon, which might result in an allergic reaction, has been employed to prolong circulation by the use of polyethylene glycol (PEGylation) surface modification [10]. It is challenging to bring liposomal drugs from the lab to the market. Controlling batch-to-batch uniformity, encapsulation efficiency, and liposome size repeatability may be challenging in large-scale production. The production of liposomal drug delivery systems is intricate and might contain multiple steps, making scalability and cost-effectiveness major challenges [11].

Clinical efficacy and regulatory approval of liposomal drugs can differ depending on these attributes [3]. Liposomal formulations are difficult to sterilize since lipids and medications that are encapsulated are sensitive to heat and radiation. The liposomes may be harmed by traditional sterilizing techniques including autoclaving and gamma radiation [12]. Although they raise the cost and complexity of manufacturing, aseptic processing and filtering are widely used. For liposomal compositions to be both economically and medicinally beneficial, encapsulation efficiency must be high [9]. Encapsulation efficiency is influenced by drug characteristics, liposome production technique, and lipid composition. It requires trial and error and patience to optimize these features. Regulation approval of liposomal composition is determined by quality and uniformity. It is important to characterize the stability, charge, encapsulation efficiency, and size of liposomes [13,14,15,16]. Some complex and costly quality control procedures arise from the use of sophisticated analytical techniques such as DLS, HPLC, and electron microscopy. Complicated regulatory pathways can be associated with complex liposomal medicines [17]. Comprehensive preclinical and clinical data are required to persuade the Food and Drug Administration (FDA) and European Medicines Agency (EMA) of the safety, effectiveness, and quality of liposomal formulations [17]. Liposomes have distinct characteristics that call for more research in comparison to conventional medications. They interact with biological systems and are biodistributed [18,19,20]. Even while liposomal formulations can improve medication delivery to particular regions, accurate targeting remains challenging. Though it complicates manufacture and design, active targeting using ligands or antibodies increases specificity. During the COVID-19 pandemic, a study showed that Amphotericin B liposomes caused increased ST-segment elevation myocardial infarction [21,22].

## 3. Strategies for Enhancing Drug Loading Capacity

For increasing the drug loading capacity in liposomes, several ways can be used to improve drug encapsulation in liposomal structures. These strategies are necessary to realize the best therapeutic potency in any liposomal drug delivery system. To acquire high drug loading capacity inside liposomes, active loading and passive loading methods have been used. Optimized preparation methods, like ethanolic injection and extrusion in liposome synthesis, attained high encapsulation and drug-loading efficiency. It was noted that the surface modification by several compounds helps to give better repulsion with the blood components, allow more drug loading capacity, and enable more circulation time [23,24].

Liposome drug loading is significantly impacted by lipid choices. Careful selection of phospholipids, cholesterol, and other lipids is necessary to create a bilayer structure that can withstand high treatment concentrations [25]. Drugs that are hydrophilic and lipophilic can be encapsulated more effectively if the chain lengths and lipid saturation are sufficient. The bilayer becomes more fluid due to unsaturated lipids. This facilitates the integration of additional drug molecules. Cholesterol molecules insert themselves between the phospholipids in the lipid bilayer by which it diminishes the movement of the phospholipid tails, resulting in a reduction in the overall fluidity of the bilayer. This mechanism guarantees that the bilayer maintains its integrity, which is essential for inhibiting premature drug release [23,26,27].

Ammonium sulfate is a good ion gradient to use in order to increase liposome medication loading. This technique actively loads ionizable medications into the liposome interior by creating an ion or pH gradient across the liposomal membrane. Once liposomes are formed in the solution, the ammonium sulfate gradient approach uses buffer instead of the external media. The pH gradient makes it possible for weakly basic medicines to accumulate, which improves liposome drug loading. Using transmembrane pH or ion gradients, remote loading, also known as active loading, encapsulates medication into prepared liposomes [28,29]. Acidic or basic medications respond best to this technique. Drugs penetrate the liposomal core efficiently by preserving a gradient. This method improves liposome stability and drug loading. Creating stimuli-responsive lipids that respond to changes in pH, temperature, or enzyme levels can enhance drug loading. These lipids undergo shape changes in response to stimulation, enabling precise and regulated medication release. pH-sensitive lipids can help load and release medications in acidic environments, such as tumor tissues, boosting the concentration of pharmaceuticals at the target site [30,31]. Polymers facilitate drug loading and liposomal stability. To increase encapsulation efficiency and circulation, polymer–lipid hybrid liposomes combine the mechanical robustness and functional versatility of polymers with the biocompatibility of lipids. Stealth liposomes are modified on the surface with polymers such as polyethylene glycol (PEG) to improve drug loading and retention while evading immune detection [32]. Drug loading in liposomes may be increased via supercritical fluid technologies, namely supercritical carbon dioxide. When lipids and drugs are mixed in scCO_2_ as a solvent, liposomes are created when the mixture depressurizes. This process is more ecologically friendly since it produces liposomes with effective encapsulation without the use of organic solvents. Freeze-drying, also known as lyophilization, increases liposomal loading and stability [33,34,35,36]. In order to extract water, this technique sublimates liposomal dispersion under low pressure. Drug loading and storage durability are improved by the concentration of freeze-dried liposomal formulation. Drug loading can be significantly increased by increasing drug–lipid electrostatic forces; liposome aggregation can be avoided and encapsulation efficiency maintained by adding cryoprotectants such as sugars or polymers during lyophilization [37,38]. Selecting lipids with charged head groups permits the lipids to engage electrostatically with medication molecules that have opposing charges. Because drug molecules are anchored in the liposomal bilayer or core by electrostatic interactions, this method is perfect for loading charged medicines [39]. By enhancing the hydrophobic interactions between drug molecules and lipid bilayers, lipophilic medicines can improve drug loading. Longer or more saturated acyl chains in lipids produce a more hydrophobic bilayer, increasing the likelihood of lipophilic drugs being incorporated. Cholesterol maintains these bonds and stops medication leaks and disintegration [40]. Drugs that are hydrophilic and hydrophobic can be co-encapsulated to increase liposomal loading and therapeutic potential. In order to achieve efficient loading and stable retention, this process entails simultaneously encasing both drug types, frequently needing adjustments to lipid composition and preparation. To accommodate the physicochemical features of pharmaceuticals, co-encapsulation can be accomplished using reverse-phase evaporation, microfluidic mixing, and thin-film hydration [24,41].

The presence of cyclic oligosaccharides, or cyclodextrins, in complexes formed by hydrophobic medicines improves their solubility and stability. Hydrophobic drug loading is enhanced by liposomal formulations that employ cyclodextrin–drug complexes. Drug loading and stability are increased when medicinal compounds are encapsulated in the hydrophobic cavity of cyclodextrins and interact with the lipid bilayer or aqueous core of the liposome [3,24,42,43,44]. Table 1 discusses the various strategies that can be used for liposome loading.

## 4. Surface Modification for Targeted Drug Delivery

### 4.1. Ligand Conjugation for Active Targeting

The targeting of liposomes is designed to be active by introducing ligands at the surface for enhanced retention at the target site and enhanced uptake by the target cells [56]. This is useful in cases where passive targeting does not occur due to the low or no existence of the EPR effect. A wide variety of ligands can be used to create tightly targeted liposomes, including antibodies that target specific surface receptors on cancer cells or the tumor microenvironment (TME) side. Proteins, such as growth factors, can target specific growth factors or cells. Peptides like Ala-Pro-Arg-Pro-Gly (APRPG) can be used to target angiogenic endothelial cells in tumors. Vitamins, such as folic acid, can target specific factors affecting cancer cells [57,58]. Growth factors like IL-4Rα can target specific neurons in the TME. Aptamers: Aptamers are single-stranded nucleic acids that can be targeted to specific receptors or cells [59,60].

In the case of antibodies, most research carried out on targeted liposomes is about using more than one antibody to target tumors. The binding of PEGylated Doxorubicin (DOX)-loaded liposomes with anti-CD22 monoclonal antibodies enhances the delivery of DOX in Non-Hodgkin’s Lymphoma xenografts, hence increasing effectiveness against tumors without increasing toxicity. A significant percentage of breast, ovarian, and stomach cancers express high levels of Human Epidermal Growth Factor Receptor 2 (HER2), while normal cells have lower levels, making it possible to target cancer therapy by ligand linkage directed at HER2. Trastuzumab, which is an anti-HER2 antibody, is also attached to paclitaxel-loaded PEGylated liposomes in order to raise their drug accumulation and increase efficacy against tumors. Additionally, a combination of a single-chain fragment antibody (scFv) against HER2 with DOX-loaded liposomes increases DOX accumulation in breast cancer tumors and demonstrates improved tumor management compared to non-targeted liposomal DOX and free DOX [61,62,63,64].

Peptide-mediated liposomes are often used to transport siRNA and anticancer drugs because they offer many benefits [65,66]. Compared to antibodies, they have a smaller and more flexible structure, making them easier to chemically synthesize for specific targeting needs. Scientists are currently exploring the use of phage display libraries to improve the targeting of nanocarriers in peptide-mediated liposomal drug delivery. While luteinizing hormone-releasing hormone (LHRH) receptors are not present in most visceral organs, they are overexpressed in cancer cells found in the breast, ovary, and prostate [67,68,69]. By attaching LHRH peptides to PEGylated liposomes, researchers have found that tumor formation and the effectiveness of anticancer treatments can be significantly enhanced while minimizing damage to healthy tissues [70]. In fact, these targeted liposomes have shown better results compared to untargeted paclitaxel-loaded liposomes. A nucleic acid aptamer called sgc8 was created by Shangguan et al. to recognize human acute lymphatic leukemia cells with extreme specificity. In an effort to determine whether the tumor-targeting IL-4Rα-aptamer liposome–cytosine-phosphate-guanine oligodeoxynucleotides (CpG ODN) delivery system could deliver CpG into tumors and circumvent immunosuppressive TME [71], Liu et al. discovered that when CT26 tumor-bearing mice were treated with IL-4Rα-liposome-CpG therapy, the anti-tumor activity was significantly higher than in the group used as a control [72].

### 4.2. PEGylation for Enhanced Circulation Time

PEGylation, or the covalent adherence of polyethylene glycol (PEG) to liposome surfaces, is a popular technique for enhancing the biodistribution and circulation duration of liposomal drug delivery systems. PEGylation creates a hydrophilic, flexible, and sterically bulky covering that prolongs blood circulation by preventing interactions with blood components and reducing absorption by the mononuclear phagocyte system (MPS) [73]. The attached PEG chain’s molecular weight (MW) has a significant role in determining the effectiveness of surface shielding. It has been demonstrated that raising the PEG MW added to liposomes (from 350 Da to 5 kDa) inhibits aggregation, adsorption to blood components, and MPS absorption, all of which lengthen circulation times. In one study, PEGylated liposomes coated with 750 Da PEG were equivalent to non-PEGylated liposomes; however, as the PEG MW was raised to 5 kDa, longer blood circulation and decreased MPS absorption were noted. PEGylated liposomes use their own extended circulation time to boost medication accumulation at the tumor site by taking advantage of the increased permeability and retention (EPR) effect in tumors [74]. PEGylation, yet, can also lessen liposome cellular uptake and endosomal/lysosomal escape, which may affect tumor retention and anti-tumor efficaciousness [75].

### 4.3. Biomimetic Approaches for Stealth Liposomes

The implementation of biomimetic materials or techniques in emulating biological structures and enhancing their activity in liposomal formulations is termed a biomimetic modification. These are various strategies for stealth liposomes as discussed further like Biomimetic Engineering of Surface PEGylation and Cholesterol-Functionalized Block Copolymers. The first method uses surface PEGylation to modify the liposomal surface, with polyethylene glycol (PEG) to develop stealth liposomes that avoid the immune system and prolong the circulation time in the bloodstream [76,77]. The latter is used to develop liposomes that self-assemble into stable nanostructures using hydrophobic and hydrophilic elements like cholesterol and PEG. This strategy uses biological membranes, giving targeted delivery [78].

Nucleolipids, a type of lipid utilized in biomimetic materials, were recently employed to create pH- and temperature-sensitive liposomes that release drugs when exposed to certain stimuli like high body temperature and acidic pH. Strategies for biomimetics, including targeted distribution of various hydrophobic and hydrophilic drugs, are possible with the help of multilamellar liposomes, which can encapsulate these compounds and improve the enhanced permeability and retention (EPR) effect [73,76,79,80].

## 5. Stimuli-Responsive Liposomal Formulations

**Figure 1:**
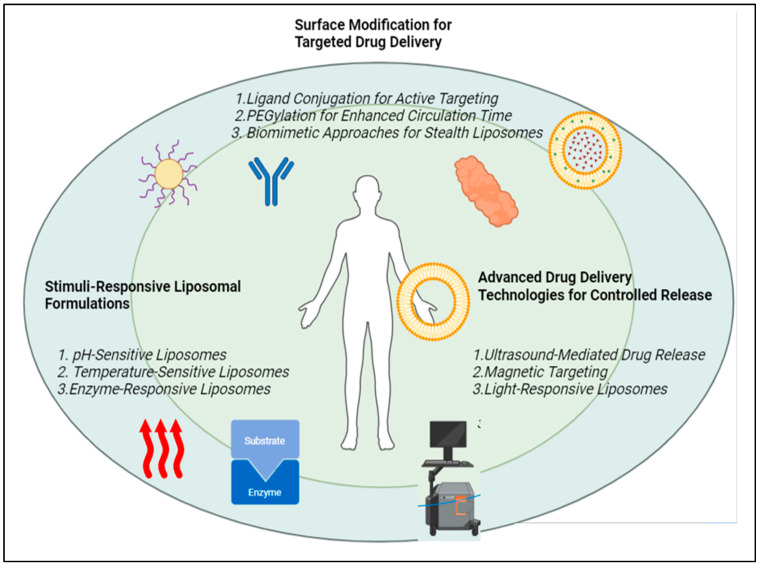
Novel methods for delivery of liposomes.

One type of nanocarrier is the stimuli-responsive liposomal formulation, which could release its payload under the regulation of specific stimuli, such as pH, temperature, light, or redox signals. The use of such “smart” liposomes could provide an improvement in the more precise and, therefore, more effective administration of drugs since they can be programmed to release their contents at a pre-assigned site or time [81]. Various novel strategies have been developed for the approach of liposomes as illustrated in Figure 1.

### 5.1. pH-Sensitive Liposomes

pH-sensitive liposomes respond to differences in pH to release their contents. One can target specific body locations, such as tumors, at places where the pH is lower than in normal tissues.

**Figure 2:**
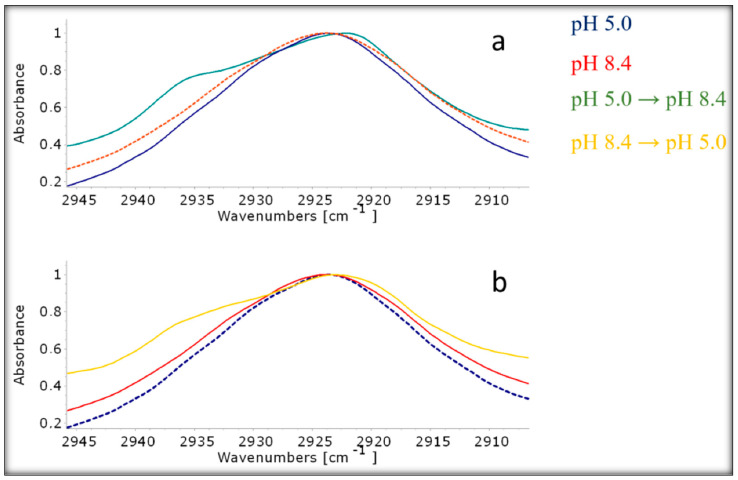
Normalized ATR-FTIR spectra of 1-palmitoyl-2-oleoyl-glycero-3-phosphocholine/ampholytic molecular switch liposomes (absorption region of asymmetric stretching vibrations of methylene groups) under various conditions: (**a**) liposomes at pH 5.0 (blue), at pH 8.4 (red dashed line), and converted from pH 5.0 to 8.4 (green); (**b**) liposomes at pH 5.0 (blue dashed line), at pH 8.4 (red line), and converted from pH 8.4 to pH 5.0 (yellow). Total lipid concentration 5 mg/mL, αAMS = 0.1, 22 °C. SEM images of *C. crispus*, *G. gracilis*, and *G. Corneum* 3D gels. Reprinted/adapted with permission from Ref. [82].

An earlier study investigated a novel type of liposome that is sensitive to changes in pH. These liposomes contain a special molecular switch called AMS, which has cationic groups and anionic groups attached to its ends. The study aimed to understand how these liposomes can quickly release their cargo. Liposomes containing AMS demonstrated rapid release of the material enclosed when the pH of an external solution was altered. The details of the rapid cargo release were obtained through atomistic chemical modeling and ATR-FTIR spectroscopic data analysis, as shown in Figure 2. The findings indicate that pH-sensitive liposomes containing AMS hold promise for medication delivery in the future [82]. The pH-sensitive liposomes guarantee effective endosomal escape since the proton sponge mediates it [83]. A study was conducted on paclitaxel-loaded pH-sensitive liposomes to evaluate the cellular absorption and release. The results showed that paclitaxel was released based on the pH with an increase in acidic pH. Although the liposomes showed cellular absorption, they also showed cytotoxicity in cancer cells. A study for melanoma treatment was conducted using daunorubicin-loaded pH-sensitive liposomes. The results showed that in melanoma cells, the liposomes had greater cellular absorption and cytotoxicity than free daunorubicin. The liposome preparation was stable at physiological pH but unstable at acidic pH for the drug to leak out [84].

### 5.2. Temperature-Sensitive Liposomes

Temperature-sensitive liposomes, a type of modern drug delivery vehicle, release their therapeutic load when the temperature varies. Phospholipid bilayers in liposomes undergo phase changes at specific temperatures. At a specific temperature, the structure of the liposomal membrane changes [85,86,87,88]. Medication release that is precise and controlled is made possible by increased permeability. Because targeted therapy necessitates precise medication delivery, this feature is helpful. Because liposomes are temperature-sensitive, they can be used for a variety of medical applications. These systems have the ability to release medication when the body temperature rises, such as during a fever, or when the tumor is heated externally to enhance drug delivery in cancer therapies. Specific lipid molecules that change from solid to liquid at a specific temperature are typically employed to produce temperature sensitivity. This smart delivery method lessens the medication’s effect on healthy tissues, minimizing side effects and increasing effectiveness. Temperature-sensitive liposomes have the potential to improve the accuracy and efficiency of drug delivery [89,90].

**Figure 3:**
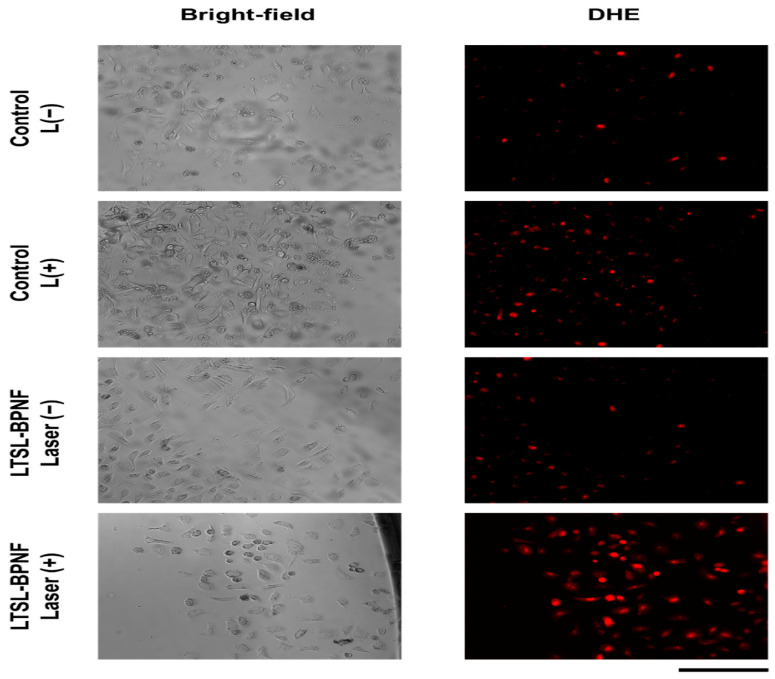
Live imaging of PC-3 cells under LTSL-BPNF therapy Reprinted/adapted with permission from Ref. [91]. After seeding 96-well plates with 2 × 10^4^ PC-3 cells each, the cells were allowed to proliferate for the entire night. The cells were exposed to a dose of 0.5 mM of LTSL-BPNFs for three hours the next day. The cells were then exposed to a 10 min laser irradiation with a power density of 1 W/cm^2^. The cells were then incubated for 30 min after the addition of 1 µM of DHE to the culture medium. After washing the cells with HBSS to remove any leftover fluorescent probe, the MuviCyte kit was used to take live cell pictures. The scale bar has a length of 500 μm. SEM images of *C. crispus*, *G. gracilis*, and *G. Corneum* 3D gels.

A study reported the production of stable and highly concentrated BP suspensions using lysolipid temperature-sensitive liposomes (LTSLs). This approach allowed for co-encapsulating Black Phosphorus nanoflakes and doxorubicin, a potent chemotherapeutic drug. BP/doxorubicin formulation revealed per se high antiproliferative action against an in vitro prostate cancer model and that the anticancer activity can be enhanced through NIR irradiance as shown in Figure 3 [91]. When there is hyperthermia, the temperature-sensitive liposomes can release their contents at a temperature of >40 °C [92,93].

### 5.3. Enzyme-Responsive Liposomes

**Figure 4:**
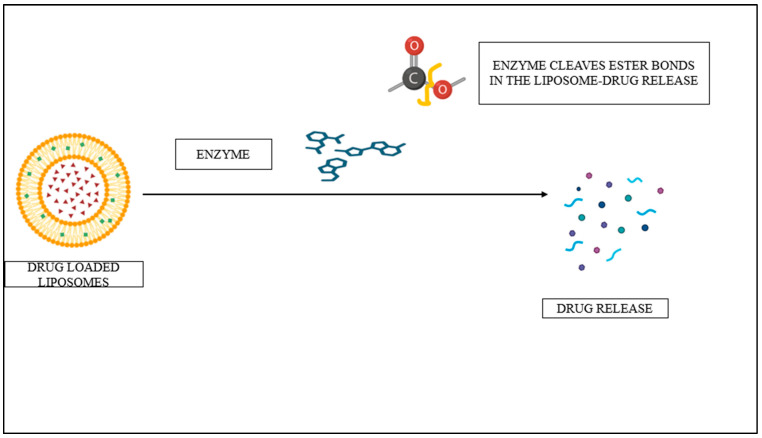
Mechanism of enzyme-responsive liposomes.

Enzyme-responsive liposomes release their contents only when particular enzymes are overexpressed in exhausted tissues such as cancer. Structural changes to the lipid bilayer occur when these liposomes are in contact with the target enzyme, the shielding polymers are removed, lipopeptides or biopolymers are cleaved, or prodrugs are activated; thus, the enclosed drug is released [81,94,95]. Thus far, researchers have prepared a general strategy for enzyme-responsive liposomes using various overexpressed targets in disease, such as esterases and other enzymes [96]. Figure 4 is an illustration of the mechanism of enzyme-responsive liposomes. Recent studies describe ongoing studies in the field like the optimization of lipid composition to improve thermal sensitivity and stability, an in vivo study to prove the effectiveness of enzyme-triggered liposomes for drug delivery and inhibition of the growth of tumors, and combining the functionality of enzyme-responsive sequences with other therapeutic compounds, such as chemotherapy and immunotherapy, to maximize their therapeutic potential and improve treatment outcomes. Much more research is yet to be conducted to translate these promising findings into the clinic. This will include making the manufacturing procedures scalable, carrying out clinical studies, and combining enzyme-responsive liposomes with multimodal imaging strategies for superior resolution and tracking of drug delivery and treatment outcomes [97].

## 6. Advanced Drug Delivery Technologies for Controlled Release

### 6.1. Ultrasound-Mediated Drug Release

Using the mechanical and thermal effects of ultrasound, ultrasonography-mediated drug launch in liposomes is a non-invasive, non-ionizing method that improves drug delivery and therapeutic efficacy. Using ultrasound-sensitive liposomes, which may be activated by freeing the medicines they include in reaction to specific ultrasound frequencies and intensities, is the mechanism used in this process. Ultrasound has essential approaches for releasing medication from liposomes. First is thermal release; it is the end result of acoustic power being absorbed, which raises the temperature locally. Drugs from thermally responsive providers, which include temperature-sensitive liposomes (TSLs), can also launch because of this temperature boom. Second is mechanical release, where cavitation and sonoporation are mechanical effects of ultrasound that can be used in this approach [98,99,100]. Thermosensitive polymers (TSPs) have been brought to liposomes to make them more sensitive to excessive temperatures in an effort to deliver capsules via ultrasound. After being incubated at 42 °C for 15 min and uncovered to 1 MHz ultrasonic radiation at 0.5 W/cm^2^ for a hundred and twenty seconds, TSP-modified liposomes launched the encapsulated calcein. Ultrasound irradiation advanced most cancer cells’ uptake of the introduced remedy. HepG2 cells treated with DOX showed higher mobile viability at 6 h after an ultrasound remedy than did cells handled with DOX-loaded TSP liposomes [101].

It has been confirmed that low-frequency ultrasound (LFUS) will increase drug release from liposomes without compromising the integrity of the substance. The lipid bilayer reports mechanical strain from the oscillating ultrasonic area, which creates fuel bubble nuclei. These gas bubble nuclei motivate temporary pore introduction and membrane breakage, which allow the payload to get away. Ultrasound-prompted release is dependent on several elements, such as the lipid composition, the presence of PEG-lipopolymers, and the coupling with metal nanostructures. To improve ultrasound-mediated medicine release, recent research has reported enhancing the chemical makeup of liposomes, specifically the phospholipid head group, and adding metal nanostructures or thermosensitive polymers. The release of liposomes is frequently caused by way of the mechanical impacts of ultrasound [102].

Many studies have been conducted on the complicated qualities of curcumin, demonstrating the extensive range of fitness benefits it offers. Its hydrophobic traits and limited bioavailability, however, create bold boundaries to its wider utility as a healing or medicinal agent. Advanced shipping techniques which are specific are vital to conquer these limitations. By combining nanoliposomes, curcumin, ultrasound, and microbubbles to improve medicine delivery and healing effects, our work affords a unique approach for most cancers’ therapy. The study elucidates the nanoscale dimensions of curcumin-loaded liposomes (CLs), thereby organizing them as an achievable candidate for scientific packages. Key additives of solid and dependable medicinal drug transport include CLs’ low polydispersity index and long-time period stability. The study efficaciously encapsulates curcumin inside CLs, as confirmed by means of studies of fluorescence depth and UV-Vis spectra. The notable improvement in fluorescence intensity indicates that curcumin’s solubility and fluorescence are greatly more advantageous through the liposomal method [102]. For drug transport structures to guarantee a steady and predictable launch of healing substances, a controlled launch mechanism is important. Ultrasound-mediated drug delivery, in comparison to standard techniques that rely upon passive diffusion, adopts an active method by using particularly targeting tumor cells, allowing for specific and controlled drug release even as it reduces the opportunity of adverse effects. Interestingly, the FDA has accepted liposomes and microbubbles as favored vendors for therapeutic use, setting them apart from different nano-providers like nano emulsions [103].

### 6.2. Magnetic Targeting

Magnetic targeting refers to the orientation of liposomal magnetic properties towards specific targeted cells or tissues. In this method, magnetic nanoparticles are entrapped into liposomal bilayers so that the magnetic field can drag the liposomes and take them to a target location.

In this way, the feasibility of synthesizing liposomes based on ultrafine magnetite is convincingly shown, as well as the possibility to probe these liposomes’ transport properties in a magnetic field both in vitro and in vivo. The latter work has been followed by extending the concept to quite different therapeutic approaches, including the application of magnetic targeting with other modalities such as ultrasound [104]. A recent study conducted with the successful fabrication of biocompatible and degradable MgZn helical micropropellers has demonstrated the capability of shape, dimensions, and composition control. These microstructures can be grown in large numbers on a two-inch wafer substrate, giving a relatively uniform complex shape despite the high mobilities of adatoms. The corrosion rate is tunable from a few hours to some weeks by adjusting the ratio of Mg: Zn, thus making it suited for diverse applications. It consists of a thin, transferred layer of hard-magnetic, biocompatible FePt in its L10 phase. It is thermally stable and can be actuated by weak magnetic fields in the low mT range. The portion of magnetic material for propulsion can be tuned to several settings depending on viscosity conditions. These might include very high viscosity conditions where high torque is required for propulsion but requires higher force to steer. Magnetic transfection experiments involve applying a magnetic field gradient that “pulls” magnetic nanoparticles toward a cell culture. Liposomes functionalized on the surface of the MgZn structures can be released in a time-dependent manner. These structures may, thus, act as microcarriers. This points towards a promising vehicle for the targeted delivery of drugs or genes, which can be integrated with the existing toolkits for magnetic actuation and transfection. More importantly, the propeller decomposition does not reduce cell viability in clinically relevant primary retinal pigment epithelial cells, even at high propeller-to-cell ratios of 20:1. The study is a first step toward enabling the biomedical application of inorganic helical magnetic micropropellers fabricated with biocompatible and degradable MgZn bodies. This material system is of interest because it promises to provide a solution to the concerns raised about the long-term biosafety of use for many potential applications in the field of gene therapy. Their application may provide a unique treatment outlook for diseases in hard-to-reach tissues where targeted, precise delivery is needed [105].

### 6.3. Light-Responsive Liposomes

The kind of nanocarrier that can release its payload in response to particular light stimuli is called a light-responsive liposome. Because this region of the electromagnetic spectrum can penetrate deeply into tissues, as opposed to other light spectra, the formulation of such liposomes has been conducted by many different wavelengths of light. Two primary mechanisms are involved in the processes of light-responsive liposomes: Photo-destabilization: The release of cargo in a destabilized liposome is shown to occur. Photo-activation: If the cargo contains photocleavable or photoprotecting groups, activation of these groups through light can release the cargo. Light-responsive liposomes have some benefits in delivery to specific places inside the body, whereby light can be used to target a particular site within the body, hence making the cargo delivery more precise. Moreover, because the cargo can be released on demand and in response to specific light stimuli, better control over the delivery processes is possible [106]. Research was conducted on protein delivery by near-infrared light-responsive liposomes. Here, they developed a platform that includes the components of gold nanorods for near-infrared light-responsiveness to distribute thermosensitive phospholipid (DPPC) and nonionic surfactant (Brij58) on demand. The technology was used to assess the urokinase-plasminogen activator (uPA) for on-demand delivery to achieve photothermally assisted thrombi [107].

## 7. Prodrug Approach Based Liposomes

Prodrug-based liposomes are an upcoming delivery option. A novel methodology has been developed. It involves modifying the molecular structure of APIs to improve their permeability and partition coefficient, improving the encapsulation. A study explored the incorporation of fructose as a polar molecule to various APIs, revealing that the effects on permeability were API-specific [108]. Another study presented a strategy combining rational prodrug design with liposomal encapsulation targeted to chemotherapeutic agent SN38. The resulting liposomes demonstrated high stability in circulation and targeted delivery to tumor sites [109].

An innovative study developed ultrasound-activated prodrug-loaded liposomes for cancer therapy. These liposomes were designed to release their drug content upon ultrasound activation, allowing for precise control over drug delivery. It showed effective cellular uptake and distribution [110].

## 8. Preclinical and Clinical Studies of Next-Generation Liposomal Formulations

### 8.1. Cancer Therapy

Through a series of engineering modifications, significant improvements have been made to enhance the stability, targeting precision, and release patterns of these advanced liposomes [110]. A notable breakthrough in the field involves the creation of liposomes that have been improved with specialized surface modifications. These modifications, including tailored ligands or antibodies, allow for targeted binding to cancer cells while reducing unintended side effects. In addition, the incorporation of stimuli-responsive elements, such as temperature- or pH-sensitive lipids, enables precise modulation of drug release based on the specific conditions of the tumor microenvironment as summarized in Table 2. A preclinical study was conducted, using mice, on Glutathione DOX–PEGylated liposomal formulation. The results showed that the Glutathione DOX–PEGylated liposomes showed better-targeted delivery than free DOX [111]. Another preclinical study was conducted on liposomal doxorubicin, using ultrasound (FUS)-induced disruption of the BBB. The study used rats, comparing DOX-FUS and free FUS. The DOX-FUS showed better delivery than FUS. Nanoparticles are an emerging technology due to their effective binding capacity and are useful in the treatment of cancer [112]. Nanoparticles encapsulated in liposomes can be one of the approaches for managing cancer [113]. A clinical study of pegylated liposomal doxorubicin and carboplatin was conducted on tolerance, response rate, time to progression, and survival in malignant uterine tumors. The study results have not yet been reported [114]. A phase 1 clinical study using vinorelbine liposomes injection(vli) for the treatment of Hodgkin’s Disease was also conducted. The study measures safety, tolerability, and the maximum tolerated dose. The results have not yet been reported [3].

### 8.2. Infectious Diseases

Next-generation liposomes have been meticulously engineered to possess unique properties and enhanced functionalities, allowing them to better tackle the challenges posed by viral infections. Several studies conducted using liposomal ampicillin showed better efficacy than free ampicillin. It was shown that splenic and bacterial counts in C57BL/Ka nude mice infected with *Listeria monocytogenes* EGD-e was greatly decreased [118]. A study conducted using a mouse animal model showed that when Liposomal Amphotericin B was given intravenously, it was sustained more than one day up to a few weeks, depending on the tissue [119]. A study was conducted for cutaneous leishmaniasis for nanoliposomal therapy to evaluate whether a complete cure or a complete re-epithelization of all lesions has occurred. The results have not yet been posted [120]. A few advanced liposomal systems are listed in Table 3 that have been checked for infectious diseases.

### 8.3. Inflammatory Disorders

Advanced liposomes have been developed to precisely deliver therapeutic chemicals to areas of inflammation, unlike older treatments that often have broad effects throughout the body and can cause unwanted side effects. These advanced liposomal systems have undergone significant design improvements as listed in Table 4. A preclinical study was conducted using prednisolone in PEGylated liposomes for the treatment of rheumatoid arthritis. It was observed that it reduced the side effects of the drug significantly [124]. Another study was conducted using dexamethasone liposome. It was observed that it showed consistent anti-inflammatory actions [125,126]. A clinical study conducted a comparison between liposomal prednisolone versus pulse methylprednisolone [127]. The results are yet to be posted.

### 8.4. Neurological Disorders

The advancement in scientific engineering holds great promise for more effective treatment of diseases such as multiple sclerosis, Parkinson’s disease, and Alzheimer’s disease. In addition, specific ligands can be incorporated into the liposome surface to modify its properties and facilitate the transport of medications through the blood–brain barrier. This targeted approach reduces the required dosage and minimizes overall side effects while enhancing drug delivery. Advancements in drug delivery have been made possible through the development of stimuli-responsive liposomes. These innovative liposomes are designed to release their contents in response to specific triggers such as changes in pH or the presence of specific enzymes [136].

A study was conducted on Parkinson’s disease patients using Talineuren liposomes to check the safety by parenteral administration. The secondary objective was to find the dose from the safety profile [137]. Another study was conducted for schizophrenia patients using liposomal glutathione to evaluate whether a higher level of glutathione was observed in the brain using this formulation [138]. Participants are being recruited for a study to evaluate the safety of ADx-001 in healthy persons. ADx-001 is a β-targeted liposomal macrocyclic gadolinium (Gd) for use in contrast-enabled MR imaging of amyloid plaques. The targeting moiety is a novel lipid—PEG-conjugated styryl-pyrimidine [139].

### 8.5. Other Therapeutic Applications

For instance, the next generation of liposomes finds application in dermal or transdermal use for enhancing the effect and stability of medicinal substances dosed to the skin [118]. Equally, they find their use within cosmetic products since these could encapsulate and distribute disparate active substances, significantly increasing their stability and bioavailability. They also have uses in food products to enhance the distribution of the bioactive components and uplift their nutrient bioavailability and stability. Besides the fact that liposomes target cells or tissues, they are also found exploring therapeutic payload delivery to the eye. Liposome applications are also carried out in nucleic acid delivery, such as mRNA, to specific tissues or cells based on treatment against different diseases [27,42].

## 9. RNA Liposomes

RNA liposomes are lipid-based nano carriers to deliver RNA. They protect RNA from degradation by encapsulating it. They majorly consist of cationic lipids that form a bilayer structure allowing encapsulation of negatively charged RNA [140]. They have several applications like, in cancer immunotherapy, mRNA vaccines and gene therapy. RNA-loaded liposomes are being investigated for their role in cancer vaccines [141]. They can deliver a modified tumor-derived mRNA, which aids in stimulating an immune response against specific cancer antigens. Lipid nanoparticles (LNPs), a category that includes RNA liposomes, have shown potential in COVID-19 vaccines [142,143,144]. RNA liposomes have been used in gene therapy, where they can deliver therapeutic RNA to correct genetic disorders or provide new functions to cells. Their ability to encapsulate various types of RNA (mRNA and siRNA) makes them a versatile therapy option [145,146,147]. The major challenge that remains for RNA liposomal delivery is to achieve targeted delivery. Further research on improving targeted delivery, optimizing liposomes, and combination therapy should be carried out.

## 10. Challenges and Considerations in Translation to Clinical Practice

### 10.1. Scalability and Manufacturing Complexity

While liposomes have shown enormous potential in the delivery of drugs, their scalability and manufacturing complexity remain a significant hindrance to being utilized clinically [16]. The process also has several hydration, homogenization, and purification steps, upscaling which can be complex due to the lack of specialized equipment and internal know-how. Also, the presence of surface modifications, coating, and ligands makes the production process complex and non-scalable. The other drawback is chemical instability due to the denaturation of the enclosed molecule in the process of making these, which is then transferred onto the liposomes, undermining the efficiency or stability of these carriers [148]. This makes it somewhat tricky later to ascertain the final products’ reliability and reproducibility, especially during large-scale manufacturing. Furthermore, the very complicated nature of the manufacturing process may increase the price of liposome production, making it difficult to use them in a clinical setting. To combat these problems, innovative manufacturing techniques for making the production process simple and easy to scale up and repeat have been developed. Examples of such strategies are solvent-free manufacturing methods and microfluidic processing [149,150,151]. Quality by design, a statistical approach using experimental design, has also been used to eliminate the challenges [152,153]. A study was conducted using a solvent-free manufacturing method for Doxorubicin-loaded PEGylated liposomes. It showed high drug loading and a low Poly Dispersity Index.

### 10.2. Regulatory Requirements and Approval Pathways

Liposomes, as one kind of nanocarrier, show potential and have received increasing attention in recent times for medicine solubility enhancement, targeted delivery, and controlled release. However, the process of approving and regulating these is complex and demanding. The process forms part of a challenging regulatory environment that governs liposomes, marked by poor regional harmonization, leading to heterogeneity in the setting of standards and acceptance criteria for pharmaceutical products based on liposomes. Another added complexity arises because liposomes have a very complex nature regarding stability and physicochemical properties; therefore, this bounds the thoroughness of their characterization, which may be pretty costly and time-consuming. Another significant hurdle in the regulatory approval process is the development of reliable in vitro release methods and biologically relevant studies to demonstrate the safety and efficacy of liposomes. Yet, although these are complex systems, even academic and commercial developers will probably feel overwhelmed by the task of presenting competent and up-to-date information regarding new liposomal therapeutic products for evaluation by regulatory agencies such as the FDA and EMEA. In general, the lack of harmonization between the different countries, the need for careful characterization, reliable in vitro release techniques and biologically relevant testing make regulatory requirements and the approval pathways for liposomes challenging [154,155].

### 10.3. Clinical Trial Design and Patient Selection

Clinical trials on liposomal formulations bring forth some challenges regarding patient selection and study design based on some characteristics of liposomes themselves, such as complex formulation and the potential for fluctuation of physicochemical features. For instance, their complexity would imply the need for some characterization of liposomes, and maybe this fact presents problems in the definition of requirements and acceptance criteria for liposome pharmaceutical products. Variations in physicochemical properties also make it challenging to design clinical trials in a way that caters to the differences. Some of these physicochemical characteristics can also contribute to the safety and effectiveness of their products. The other challenge at the time of the regulatory approval process is the call for robust in vitro release methods coupled with biologically relevant data to ascertain the safety and effectiveness of the liposome. Since liposomal formulations are most of the time prepared with some populations in mind, patient selection is very crucial. On the other hand, patient selection for suitable patients can be considered an essential aspect that is hard to refine, considering the complexity of the required characterization of the properties of liposome-based drug delivery systems [156,157].

## 11. Future Perspectives and Emerging Trends

The development of modern liposomal systems with enhanced biocompatibility, biodegradability, and targeted transport skills has been facilitated by the latest traits in liposome generation. The development of an increasing number of intricate and superior structures that could cope with the difficulties of managed release, extended lifetime, and intracellular shipping is what will shape the future of liposomal drug shipping systems. Furthermore, it is predicted that the combination of nanotechnology and biomaterials could enhance liposomal structures’ therapeutic efficacy and safety even in addition. Nanoparticles and theranostics are emerging trends, and liposomes can be used as strategies for treatment using these trends [155,158].

## 12. Conclusions on Implications for Future Research and Clinical Practice

Liposomes have emerged as a versatile and promising device for cantered drug delivery, supplying significant healing functionality in numerous illnesses. The improvements in liposomal methodologies, commercial formulations, and medical trials have tested their efficacy and protection in treating various cancers, infectious diseases, and specific conditions. However, there is nevertheless a need for further studies to optimize liposomal formulations, enhance their biocompatibility and biodegradability, and beautify their targeted transport abilities. Future studies must be recognized for developing more sophisticated liposomal systems that could deal with the demanding situations of controlled launch, prolonged lifetime, and intracellular delivery. Additionally, there may be a need for additional complete clinical trials to set up the long-term protection and efficacy of liposomal formulations.

## Figures and Tables

**Table 1:** Different strategies for liposome loading.

Strategy/Method	Description	Remark	Ref
Passive Loading	Drugs are encapsulated during liposome formation, typically through thin-film hydration or ethanol injection methods.	**Limited by Lipid Solubility:** Efficient for lipid-soluble drugs but less so for water-soluble drugs.	[24]
Active (Remote) Loading	A transmembrane pH or ion gradient is created to drive drug molecules into preformed liposomes.	**High Loading Efficiency:** Works well for weakly basic drugs, achieving high drug-to-lipid ratios.	[45]
Use of Cholesterol	Cholesterol is incorporated into the lipid bilayer to modulate membrane fluidity and drug encapsulation.	**Increased Stability, Variable Capacity:** Stabilizes the membrane but can reduce the volume available for drugs.	[24]
PEGylation (Polyethylene Glycol Coating)	Liposomes are coated with PEG to improve circulation time and potentially increase drug loading.	**Extended Circulation, Limited Effect on Loading:** Enhances liposome lifespan but does not directly increase loading.	[46]
Lipid Composition Alteration	Adjusting the lipid composition (e.g., using cationic or anionic lipids) to enhance drug encapsulation.	**Improves Loading for Specific Drugs:** Works best for drugs with charge compatibility with the chosen lipids.	[47]
Inclusion of Charged Molecules	Incorporating charged lipids or surfactants to improve drug encapsulation, especially for ionic drugs.	**Effective for Ionic Drugs:** Enhances encapsulation efficiency for oppositely charged drugs.	[48]
Solvent-Assisted Loading	Use of co-solvents (e.g., ethanol) to enhance drug solubility during liposome formation.	**Improves Solubility, Limited by Solvent Type:** Can increase drug loading but residual solvents need removal.	[49]
Double Emulsion Method	A water-in-oil-in-water (W/O/W) emulsion is used for the encapsulation of hydrophilic drugs.	**Improved Hydrophilic Drug Encapsulation:** Suitable for encapsulating water-soluble drugs, but complex to control.	[50]
Proliposome Approach	Dry lipid formulations that form liposomes upon hydration, potentially improving drug loading during formulation.	**Convenient for Large Scale, Variable Efficiency:** Can enhance loading for certain drugs, but results vary.	[51]
Temperature Gradient Method	A temperature gradient is applied to increase drug solubility during liposome formation.	**Useful for Temperature-Sensitive Drugs:** Can improve loading but may not work for temperature-sensitive compounds.	[52]
pH-Sensitive Liposomes	Designed to release drugs in response to pH changes, facilitating higher drug loading under certain pH conditions.	**Controlled Release, Loading Dependent on pH Range:** Loading is optimized for drugs that respond to pH shifts.	[53]
Size Optimization of Liposomes	Reducing liposome size via sonication or extrusion to improve drug encapsulation efficiency.	**Enhanced Surface Area, Stability Concerns:** Can enhance drug loading but may reduce liposome stability.	[24]
Use of Cyclodextrins	Cyclodextrins are included to form drug complexes, enhancing solubility and loading in liposomes.	**Improves Solubility for Poorly Soluble Drugs:** Effective for increasing lipophilic drug loading.	[54]
Freeze-Drying (Lyophilization)	Liposomes are freeze-dried with cryoprotectants to preserve them and improve drug encapsulation stability.	**Stabilization, Minimal Effect on Loading:** Primarily for stability, with limited direct impact on drug loading.	[38]
Microfluidic Approaches	Use of microfluidics for controlled mixing during liposome formation, potentially increasing loading efficiency.	**Precise Control, Scalability Issues:** Allows fine-tuned drug loading but may be hard to scale up.	[55]

**Table 2:** Preclinical study and clinical study for cancer therapy.

S. N.	Study	Animal	Disease	Type	Phase	Reference
1.	Pegylated Liposomal Doxorubicin (PLD) and Carboplatin	-	Malignant Gynecologic Tumour’s	Clinical	II	[115]
2.	Vinorelbine Liposomes Injection	-	Advanced Solid Tumors, Non-Hodgkin’s Lymphoma or Hodgkin’s Disease	Clinical	I	[116]
3.	Glutathione DOX–PEGylated liposomal formulation	Mice	Brain Cancer	Preclinical	-	[111,112]
4.	Ultrasound (FUS)-induced disruption of the BBB after the administration of liposomal doxorubicin	Rats	Brain Cancer	Preclinical	-	[117]

**Table 3:** Represents preclinical study and clinical study for infectious diseases.

Sr. No	Study	Animal	Disease	Phase	Type of Study	Ref
1.	Liposomal Amphotericin B	Mice	Antifungal	-	Preclinical	[119]
2	Liposomal Gentamicin,	Mice	Salmonella Dublin infection	--	Preclinical	[121]
3	Liposomal Streptomycin	Mice	Intracellular *B. canis*, *B. abortus* and *B. melitensis* infection of liver and spleen	--	Preclinical	[122]
4	Nanoliposomal Meglumine Antimoniate (Glucantime) or Paromomycin in Combination with Systemic Glucantime	---	Cutaneous Leishmaniasis	Early I	Clinical	[122,123]

**Table 4:** Advanced liposomal systems for inflammatory disorders.

Sr. No	Study	Animal	Disease	Phase	Study Type	References
1.	Prednisolone in PEGylated liposomes	Mice	Rheumatoid Arthritis	-	Preclinical	[128,129]
2.	Dexamethasone in liposomes	Mice	Rheumatoid Arthritis	-	Preclinical	[130]
3.	Methotrexate in Folate Tagged Liposomes	Mice	Rheumatoid Arthritis	-	Preclinical	[131]
4.	Liposomal prednisolone vs. pulse methylprednisolone	-	Rheumatoid Arthritis	II	Clinical	[132]
5.	Cyclosporin A PEGylated liposomes	Male Wistar rats	Infarct size, brain edema, and neurological activities	--	Preclinical	[122]
6.	Prednisolone PEGylated liposomes	male LEW/HanHsd rats	Renal ischemia and reperfusion injury	---	Preclinical	[133]
7.	Interleukin (IL)-10 conjugated liposomes	Obese C57Bl/6 mice	TNF-α secretion	--	Preclinical	[134]
8.	FK506 PEGylated liposomes	7-week-old male Lewis rats	Myocarditis	--	Preclinical	[135]
